# Synaptic tau: A pathological or physiological phenomenon?

**DOI:** 10.1186/s40478-021-01246-y

**Published:** 2021-09-09

**Authors:** Miranda Robbins, Emma Clayton, Gabriele S. Kaminski Schierle

**Affiliations:** 1 Department of Chemical Engineering and Biotechnology, University of Cambridge, Cambridge, UK; 2 Department of Clinical and Experimental Epilepsy, University College London, London, UK

**Keywords:** Plasticity, Tau, Synapses, Memory, Alzheimer’s disease, Neurodegeneration

## Abstract

In this review, we discuss the synaptic aspects of Tau pathology occurring during Alzheimer’s disease (AD) and how this may relate to memory impairment, a major hallmark of AD. Whilst the clinical diagnosis of AD patients is a loss of working memory and long-term declarative memory, the histological diagnosis is the presence of neurofibrillary tangles of hyperphosphorylated Tau and Amyloid-beta plaques. Tau pathology spreads through synaptically connected neurons to impair synaptic function preceding the formation of neurofibrillary tangles, synaptic loss, axonal retraction and cell death. Alongside synaptic pathology, recent data suggest that Tau has physiological roles in the pre- or post- synaptic compartments. Thus, we have seen a shift in the research focus from Tau as a microtubule-stabilising protein in axons, to Tau as a synaptic protein with roles in accelerating spine formation, dendritic elongation, and in synaptic plasticity coordinating memory pathways. We collate here the myriad of emerging interactions and physiological roles of synaptic Tau, and discuss the current evidence that synaptic Tau contributes to pathology in AD.

**Supplementary Information:**

The online version contains supplementary material available at 10.1186/s40478-021-01246-y.

## Introduction

This review is primarily focused on synaptic Tau in Alzheimer's disease. Studies that investigate the function of synaptic Tau have been obtained through various experimental models including overexpression of proteins, Tau with FTLD. We are aware of the current limitations of various animal models as they may not fully replicate human AD, however there is still much we can learn from these models especially when it comes to a molecular understanding of the disease, and we will therefore discuss results from animal models alongside results from tissue studies of AD patients.

## An overview of Alzheimer’s disease, the most common form of dementia

Alzheimer’s disease (AD) is the most common form of dementia affecting 50 million people worldwide in 2018, a number predicted to triple by 2050 to affect over 152 million people. Alongside this, 25% of hospital beds in the UK are occupied by people aged over 65 and suffering from dementia (Alzheimer’s Research UK). Age is the greatest risk factor for developing the sporadic form of AD [82, 119, 176, 184], however there are also genetic and environmental risk factors [120, 217, 219] that contribute to familial or sporadic forms of AD, respectively. The pathophysiology of AD is complex and not fully understood as we will see through the course of this review article*.*

AD is diagnosed histologically in *post mortem* brains of patients by the presence of two types of aggregated proteins with little understanding of how these proteins interact with each other during the different stages of disease. Extracellular plaques of Amyloid-beta (Aβ) peptides and intracellular neurofibrillary tangles (NFTs) of microtubule-associated protein Tau (MAPT; Tau) are both hallmarks of AD. Due to the presence of these different protein pathologies in AD, the field has been divided for a long time between people believing in either Aβ or Tau being causal to AD pathology [322]. For example, scientists who suggested that Aβ was causative of AD thought that Tau and other pathology were secondary to the cascade triggered by Aβ [172].

In the past years though, some research has moved away from preventing Aβ pathology towards inhibiting Tau pathology as the distribution and density of Aβ positive plaques are variable between neuropathological stages of the disease and not informative of the cognitive status of the patient [144]. Cognitive decline is most closely associated to the load and progression of NFTs as compared to Aβ pathology [171, 326]. Therefore, Aβ may thus be considered as a catalyst of Tau pathology [35], with Tau being a more central player in AD progression. The latter is supported by the fact that there are currently more than 20 different Tauopathies [395]. Tau pathology spreads anterogradely and follows the disease progression, the so called ‘Braak stages’, which progress from I–IV based on brain regions burdened by NFTs. Although the locus coeruleus has previously been suggested to be the starting point [41, 43, 427], recent evidence suggests that Tau pathology begins in the transentorhinal/entorhinal regions [220]. Thus, symptoms of AD highly correlate with the progression of Tau pathology from the hippocampus to the cortex, beginning with memory dysfunction and later leading to other cognitive impairments including loss of executive functioning, language, and visuospatial skills [101, 144, 245, 362]. The hippocampus is an anatomical region of the brain responsible for spatial or contextually-based learning and memory and it is one of the earliest and most drastically affected areas, displaying atrophy, accumulation of Aβ plaques, and NFTs in AD [16–18]. The role of hippocampal neuron subtypes in learning and memory is defined by their characteristic calcium dynamics, a high degree of plasticity and the capacity to undergo synaptic remodeling into adulthood. It has also been believed that hippocampal neuron subtypes are a major source of human adult neurogenesis [4, 95, 233, 498] until recent controversy [420]. The properties of hippocampal neurons are thought to impart the selective vulnerability of these cells, as pathology drastically accelerates on reaching neurons in this region at early stages of AD [156].

### Microtubule-associated protein tau (MAPT)

Full-length monomeric forms of Tau have long been seen as the ‘glue’ that binds and stabilises microtubules in axons, in concert with other microtubule-associated proteins, such as MAP2, which have homologous roles in neuronal dendrites [468]. Microtubule stability is important for cellular polarity and for antero- or retrograde cellular transport of vesicles and organelles to occur. However, as the full interactome of Tau is revealed, the ubiquity of Tau’s roles is being uncovered to show how Tau binds to a diverse range of molecules to elicit a multiplicity of functions. Before binding to microtubules, Tau is an intrinsically disordered protein which confers conformational and functional flexibility. Numerous Tau binding partners with diverse cellular functions have now been reported. Tau binds directly to DNA for DNA protection [58, 279, 432, 467], to calmodulin to regulate gene expression [24], at the cell membrane to support growth processes [257], to Fyn for synaptic activity [200, 338], to actin for crosslinking actin filaments [56] and to numerous other proteins with yet unknown functional consequences [276]. Missense mutations in *MAPT*, the gene coding for Tau*,* can result in familial forms of frontotemporal dementia but are not causative of AD [111, 197, 356]. The ability of Tau to bind and interact with such a diverse range of molecules, and thus taking up so many roles, stems from Tau being produced as six different splice variants [158], from its ability to be post-translationally modified, its diverse binding regions, and from it being prone to terminal truncations (for review see [3].

### The complex structure of tau

Six isoforms of Tau are present in the adult human central nervous system, although Tau occurs as a larger isoform in the peripheral nervous system [153].

**Fig. 1:**
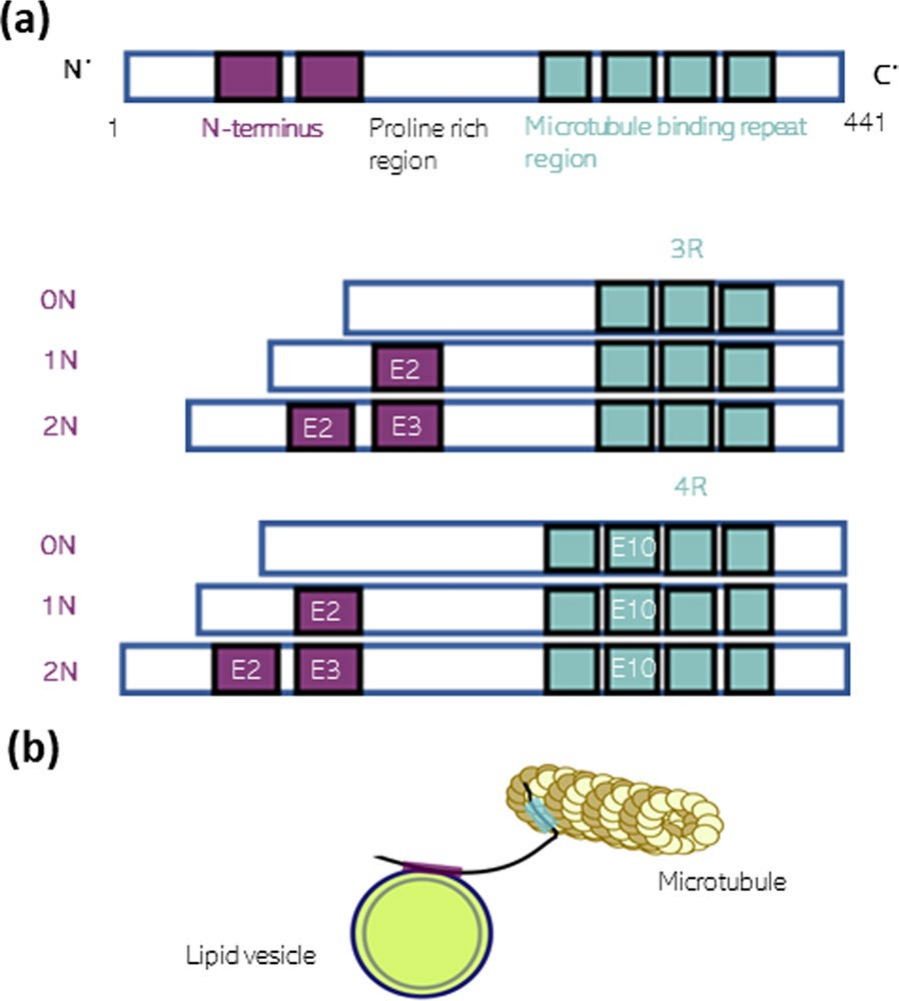
Tau is differentially expressed as six isoforms that contain multiple structural domains for diverse protein–protein interactions. **a** Alternative splicing of exons 2 and 3 (E2, E3) determines the N-terminus region, whereas exon 10 (E10) determines the number of repeat (R) regions and gives rise to 3R or 4R Tau. Overall six isoforms of Tau exist with their expression dependent upon age and anatomical location. **b** The N-terminus region of Tau is involved in membrane interactions and has been shown to bind to synaptic vesicles [44, 265, 301]. The microtubule binding repeat regions bind and stabilise microtubules [260]

Figure 1a shows how the six different Tau isoforms arise from alternative splicing. N-terminus inserts, Exons 2 and 3, result in 0 N, 1 N or 2 N Tau, whereby exon 3 is never inserted independently of exon 2. Exclusion or inclusion of the microtubule binding repeat region (MTB), exon 10, results in 3 repeat (3R) or 4 repeat (4R)-Tau, respectively, altogether providing 0N3R-, 0N4R-, 1N3R-, 1N4R-, 2N3R-, 2N4R- Tau [10, 150, 151, 260]. The N-terminus projection region has been found capable of binding to synaptic vesicles, either through protein binding (Fig. 1b, [424, 503] or through direct membrane interactions [44, 265]. The proline-rich region and microtubule binding domain are capable of polymerising F-actin, a cytoskeletal protein that has various roles in neurons including remodelling dendritic spines upon synaptic stimulation [136, 177, 194]. The proline-rich region is also able to bind SH3 domains such as Fyn kinase, which is of interest for a post-synaptic role of Tau [371]. The microtubule binding repeat region, alongside binding and stabilising tubulin, can also bind to lipid membranes [142], and part of this region forms the core of aggregates [128, 129].

Debates about the role of post-translational modifications and truncated forms of Tau are ongoing. In one study, it was shown that the major form of Tau in the pre-synaptic compartment is truncated at its C-terminal and therefore lacks the (aggregation-determining) microtubule binding domain region [416]. The release of this truncated form of Tau is increased upon synaptic activity [216]. However, a study investigating the level of truncation in AD patient brains has shown that there is a relative increase in N-terminal truncations of Tau in AD patients as compared to controls [505]. The longest isoform of Tau has a total of 85 possible phosphorylation sites that interact with multiple kinases and phosphatases [150, 151]. Phosphorylation can determine the conformation and protein–protein interactions of this intrinsically disordered protein and therefore the residues at which Tau is phosphorylated can differ between physiological versus pathological conditions (for a review see [209]. For example, phosphorylation can modulate binding dynamics of Tau to tubulin, biolipid, and Fyn kinase [200, 269, 317, 394] and thus forms part of Tau’s physiological role. For binding to microtubules, consecutive phosphorylation of Tau is required, one to allow efficient phosphorylation of the second ‘primed’ site [152]. However pathologically, hyperphosphorylation of Tau has been associated with the formation and growth of neurofibrillary tangles, as specific phosphorylation sites have been shown to readily enhance fibril formation [98].

### Aggregation of tau

In the characteristic pathway leading to the formation of neurofibrillary tangles in AD, hyperphosphorylated monomeric Tau forms small soluble granular structures known as oligomers. These oligomers have been suggested to act as toxic species which form part in AD pathogenesis [55, 284, 345, 452]. Oligomers are seen as an intermediate structure, capable of inducing a conformational change in monomeric Tau which is then able to attach to the oligomeric structure. The latter then leads to the formation of stacked β-sheet-rich strands which grow to form insoluble paired helical filaments (PHFs) which consequently amalgamate into large NFTs [129, 164, 250]. It has been shown that PHF-like Tau can lead to loss of synaptic contacts which is known to occur in hippocampal neurons of hibernating animals but reversed upon awakening [15]. Hyperphosphorylation of Tau has also been reported to occur in mice suffering from hypothermia during anaesthesia [351]. Thus, the pathogenic versus protective role of larger, insoluble structures, that are less neurotoxic than soluble oligomers but confine intracellular space and prevent intracellular trafficking [213] as they grow in size, is still an ongoing debate in the field.

### Pathological tau

How oligomeric Tau forms is still unclear. Potential pathways leading to Tau oligomerisation include Tau release from microtubules, poly-anionic induction factors, or uptake into low pH compartments [21, 231, 307]. As neuronal activity has been shown to increase the rate of Tau pathology [476], it is possible that activity-dependent pathways may also mediate its aggregation. This could be an age-dependent mechanism whereby neuronal activity over time causes the formation of pathological Tau species, and their propagation. Alternatively, high levels of network activity [355], lysosomal dysfunction [315], or cell death such as induced by traumatic brain injury [320, 376] may result in a higher concentration of Tau being released into the extracellular space. The latter then leads to endocytic uptake and aggregation of Tau at low pH [307]. Tau has been shown to cause membrane disruption allowing it to leak from endo/lysosomes [57]. It is also thought that the impaired endosomal sorting complex required for transport (ESCRT) III protein activity permits a leakage of Tau from endo/lysosomes into the cytosol [68]. Selectively, vulnerable cells may act as the primary site of aggregation. Tau released by these cells may consequently be propagated along synaptically-connected networks whereby they recruit endogenous Tau and result in AD symptoms only after several years in the brain of an AD patient [26, 134, 135, 167, 183, 226, 347]. Tau uptake through muscarinic receptors can alter calcium ion (Ca^2+^) homeostasis [157]. Many of the Tau uptake mechanisms are further increased upon phosphorylation [218, 304] or neuronal activity, which, along with how synaptic pathological Tau can perturb activity, will be discussed in the next sections [246, 303, 416, 462]. Tau is also able to form membrane pore-like amyloid structures (annular protofibrils) similarly to those seen by α-synuclein and Aβ, which have been suggested to allow uncontrolled release of aggregates, ions, or vesicles [50, 105, 252, 253, 343].

Oligomeric forms of Tau have been shown to impair synaptic function, the latter being an early marker preceding fibril formation, synaptic loss, axonal retraction and cell death [123, 241, 284, 297, 345, 353, 491]. Tau is also present at lower concentrations in the somatodendritic compartments, often considered as the loss of its physiological function as it requires the detachment of Tau from microtubules [200, 338]. Tau has previously been found in pre- and post- synaptic compartments of healthy human volunteers and AD patients, but in AD patients it is primarily found in its ubiquitinated and phosphorylated form [124, 438]. Pre- and post- synaptic forms of Tau pathology have been described without a clear mechanistic link between the two [200, 353, 503]. Since Tau is able to accelerate spine formation and dendritic elongation, and is involved in memory pathways [230, 391, 392, 496] it has recently been discussed whether Alzheimer’s disease may be described as a physiological to pathological shift of synaptic Tau function [200, 301].

### Activity-dependence of tau pathology in the hippocampus

The release of soluble Tau from neurons, both in vivo and in vitro, can be regulated by neuronal activity, and is suggested to be a physiological process. It is not known whether Tau released by neurons is monomeric or oligomeric [355, 476, 484]. Wu et al. [476] investigated whether neural activity could increase the rate of the progression of Tau pathology by increasing the activity-dependent release of Tau to synaptically-connected neurons. To test this hypothesis, cells that expressed mutant P301L hTau aggregates were stimulated with picrotoxin and approximately 45% of the stimulated cells were shown to have internalised Tau as compared with 20% of unstimulated cells. Similar results were seen in vivo, where hippocampal cells that were optogenetically stimulated for 20 days showed greater accumulation of Tau in cell bodies, and increased hippocampal cell layer atrophy compared to unstimulated animals [476]. The study did, however not include experiments to link increased pathology with behavioural deficits related to AD to see whether neuronal stimulation and the Tau pathology it induced also caused an earlier or more pronounced behavioural phenotype. From the study above, it was also unclear whether neuronal stimulation was driving Tau seed formation or whether it only increased their propagation through synaptically connected cells.

The direct relationship between neuronal activity and Tau pathology still needs to be determined. From recent research it seems likely that there is a feedback mechanism whereby neuronal activity causes increased Tau pathology, which in turn alters neurotransmission, and feeds forward to further Tau aggregation and propagation. Interestingly, Amyloid β (Aβ) induced hyperexcitability has also been linked to catalysing Tau pathology [378]. Bright et al. [46] showed such a relationship that includes a link with Aβ production. Neuronal hyperactivity, which is able to regulate increased Tau translation and extracellular Tau secretion [235, 355], has been shown to increase Aβ production. Both Aβ and Tau have been related to neuronal hyperexcitability, and Tau has been linked to pro-convulsive effects [49, 53–55, 100, 175, 187, 200, 244, 311, 339, 374, 375]. The high frequency activity that occurs in the hippocampal formation for learning and memory, as for other activities, such as spatial exploration or sleep for example, may explain an increase in pathology reaching these networks and thus the increased vulnerability of hippocampal cells. However, studies showing network hypoactivity also exist [55, 290] and therefore more research is required to reconcile the role of Tau on neuronal activity and how this may affect memory impairment during the course of AD.

## Relating tau pathology to models of memory impairment

Synapses were first hypothesised to be the primary site of memory simultaneously with their discovery by Ramón y Cajal (1894). The most well established model for activity-dependent synaptic strengthening was discovered when Lømo [278] found evoked responses to high frequency stimulation in the hippocampus that lasted for hours. Certain forms of neuronal activity, including the high frequency stimulation used by Lømo [278], result in the influx of Ca^2+^ ions into synapses. Ca^2+^ ions act as a 2nd messenger for phosphorylation-dependent signalling cascades, causing neurotransmitter release, structural plasticity of the cytoskeleton, and the incorporation or alteration of ion channels and their subunits. These alterations ultimately feedback to maintain an increased and sustained Ca^2+^ conductance and is known as long-term potentiation (LTP). Alongside LTP, its counterbalance that is induced by low frequency stimulation to decrease conductivity of synapses, long-term depression (LTD), was also discovered [423]. LTP and LTD have been heavily studied in the hippocampus where they may underlie declarative learning and memory [308, 312, 76]. Impairment to hippocampal-dependent memory function is seen as early symptom of AD, and correlates with Tau pathology in the hippocampus [16, 17, 42].

**Fig. 2:**
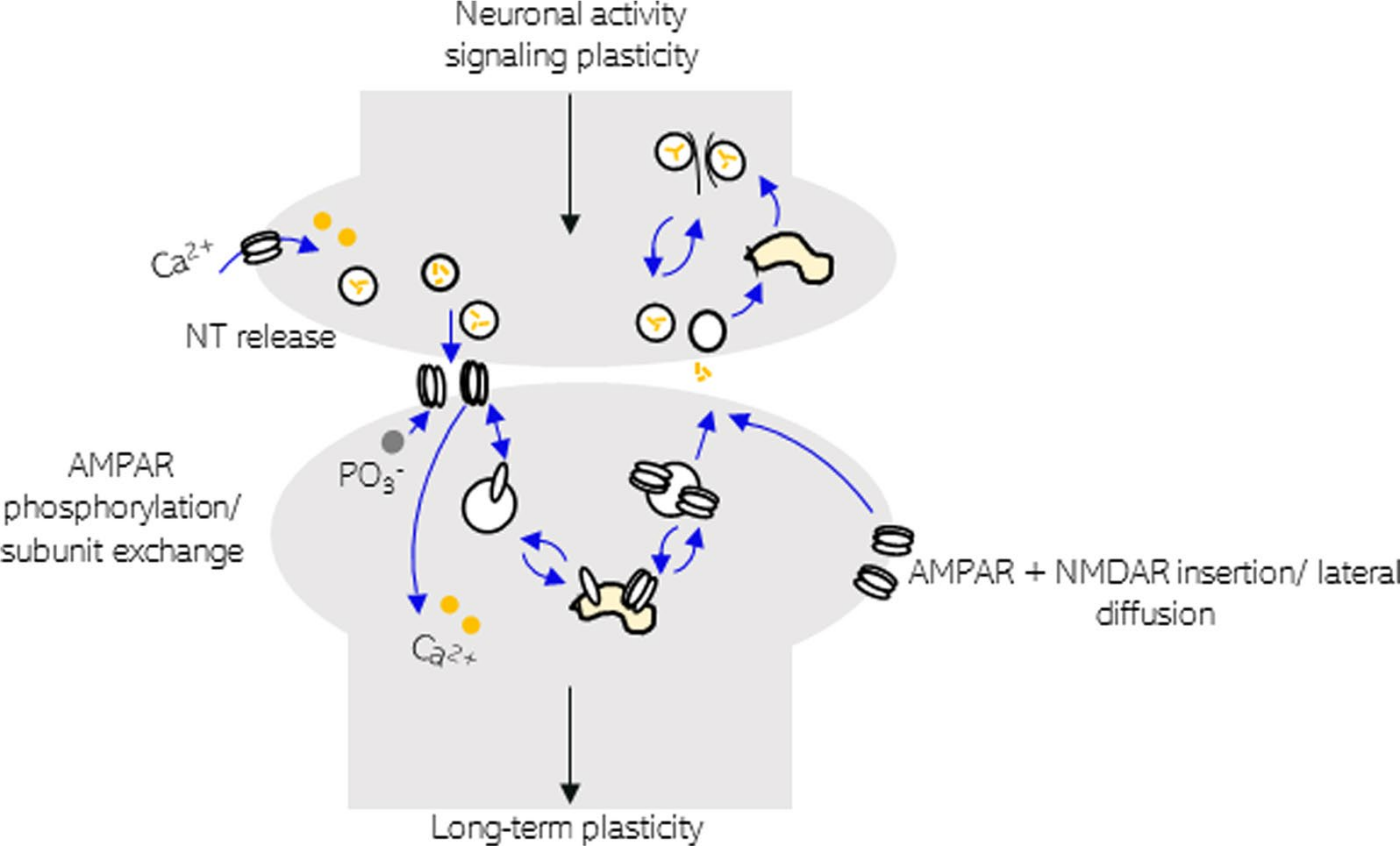
Key processes by which Tau could interfere with LTP induction to directly cause symptoms of memory impairment. Processes include channel activation or gating function for Ca^2+^ ion entry, pre-synaptic vesicle cycling, post-synaptic vesicle cycling for subunit exchange and channel insertion, recruitment of receptors tethered at extra synaptic sites, and cytoskeletal restructuring to coordinate these mechanisms and to control the synaptic volume

The next question that had to be addressed was which molecular mechanisms had occurred to maintain the enhanced synaptic response during LTP? Though a controversial field, three mechanisms have consistently shown to be important for the induction of LTP (Fig. 2). (1) The pre-synaptic mechanism increases the probability of neurotransmitter release by upregulating the number of release sites, or the concentration of cleft glutamate. (2) Post-synaptic mechanisms increases the single-AMPA receptor-conductance on binding glutamate, either by increasing their opening probability, or prolonging their mean open-time through phosphorylation or exchange of subunits. (3) An additional post-synaptic mechanism increases channel numbers by inserting receptor-containing vesicles into the plasma membrane, or by lateral diffusion of extrasynaptic regions [34]. The reversal of these mechanisms can instigate LTD. It needs to be noted here that both, LTP and LTD remain a means to model memory, and do not necessarily equate to human hippocampal memory. However, the above mechanisms involve cytoskeletal restructuring for controlling synaptic volume, for stabilising active zone synaptic densities, and for cycling and tethering of vesicles or proteins via cell membrane endo- and exocytosis or via recycling vesicles. Increased import of proteins into synapses, or local translation [84, 215], is also required. As the latter mechanisms are involved in memory formation, we thus think that LTP and LTD are a relevant model to study certain aspects of memory formation. Indeed, evidence that Tau can influence any of these mechanisms, either physiologically or in pathological conditions, would provide a direct molecular to behavioural link of how Tau may lead to memory impairment.

Tau may directly influence LTP and LTD. Tau knockout ameliorates Aβ induced deficits in LTP. Aβ oligomers show a fairly consistent impairment in LTP and enhancement of LTD [249, 402, 460]. Endogenous wild-type Tau expression, but not the N296H FTLD mutant form, is required for Aβ dependent impairment of LTP [453], and impairment of LTP by Tau or Aβ oligomers is dependent upon amyloid precursor protein expression [360, 464].

However, studies have shown varying effects dependent on age, disease model, and protocol used when understanding how Tau may alter electrophysiological properties of cells. Table 1. summarises how the effects of Tau on electrophysiological measurements have a large degree of variability depending upon the methods applied. The most consistent finding appears to be that exogenous oligomeric Tau impairs LTP, with monomeric Tau having no effect [123, 251, 352, 360, 434]. Fá et al. [123] showed that a 20 min treatment of CA3-CA1 hippocampal neurons with oligomeric 4R2N Tau before induction of LTP caused a marked reduction in LTP expression without affecting basal synaptic transmission. On the contrary, 4R1N monomeric Tau did not reduce LTP. Polydoro et al. [352] predicted that Tau impairs the induction rather than the expression of LTP as high frequency but not theta burst stimulation failed to induce LTP in a hTau mouse model. Another study in rTgP301L mice expressing mutant Tau showed an impairment to both basal transmission *and* LTP [190] consistent with two similar studies using P301S or hTau mice [352, 491]. One study has even shown improved cognitive performance and LTP in the dentate gyrus of young Tau-P301L mice, and suggested hyperphosphorylation of Tau to be the pathogenic cause of synaptic impairment [37]. Many other studies have also linked Tau pathology to poor cognitive performance at a behavioural level [14, 205, 365, 393, 435, 440], and the suppression of Tau expression with an amelioration of symptoms [391, 435].

**Table 1:** The changes to basal transmission and LTP measured in different mouse models expressing endogenous mutant, human or wildtype Tau. Results show the large amount of variation dependent upon the method applied, but exogenous oligomeric Tau is consistently impairing LTP

Study	Model and Tau expression	Basal transmission	LTP
Boekhoorn et al. [37]	9-week Tau-P301L mice. 2 × expression level as compared with endogenous Tau (controlled for in wildtype); Under Thy1 promoter	No change	Increase
Schindowski et al. [393]	G272V and P301S (Thy22) mice. 4–sixfold expression level as compared with endogenous Tau; Under Thy1.2 promoter	Reduced	No change
Hoover et al. [190]	TgP301L mice. ∼13-fold-expression level as compared with endogenous Tau; Under CaMKII promoter	Reduced	Impaired induction
Yoshiyama et al. [491]	P301S (PS) mice. 3–fivefold expression level as compared with endogenous Tau (controlled for in wildtype); Under mouse prion (MoPrP) promoter	Reduced	Impaired induction
Polydoro et al. [352]	hTau mice. Expression not determined but higher than endogenous levels; Under Tau promoter	Reduced	Impaired
Koch et al. [236]	Human AD patients	N/A	Impaired. Reversal of LTP toward LTD
Fá et al. [123] Lasagna-Reeves et al. [251] Puzzo et al. [360]	Oligomeric exogenous Tau and wildtype mice	No change	Impaired
Maeda et al. [284]	hTau-A152T mice. Three–fivefold expression level as compared with endogenous Tau; Under CaMKII‐tTA promoter	Increased	No change
Decker et al. [92]	hTau- A152T mice	Increased	No change

In the studies listed in Table 1, no attempt was made to explain the molecular mechanism of how Tau impaired LTP. The relationship between Tau pathology and these activity-dependent mechanisms (Fig. 2) therefore requires further explanation. Very different results for how different forms of Tau can alter the electrophysiology of neurons can be seen in Table 1. One suggestion for the variation between models is the location and concentration of Tau expression, and the mutation site for the different forms of mutant Tau used. An example of mutations in different domains resulting in opposite electrophysiological functional effects is A152T [242] at the N-terminus projection domain, versus K280del in the second microtubule-binding repeat domain [316, 373]. A152T expressing mice show increased basal transmission with increased glutamate release, without changes to synaptic plasticity [92]. Mice overexpressing K280del show reduced basal transmission with reduced pre-synaptic vesicles, and impaired synaptic plasticity [91]. This, however, does not address how wildtype Tau in Alzheimer’s disease functions. An additional cause of variability may be when different Tau isoforms contribute differentially to pathology, though the relationship is unlikely to be so simple, for example 0 N and 1 N Tau result in similar electrophysiological phenotypes (Table 1, [190, 491, 503]. Different isoforms of Tau have different roles in dendrite and spine formation, and it has been argued that the pathological mis-sorting of Tau, from the axon, is dependent on the level of specific Tau isoforms, though this may also just be driven by overexpression [444, 496]. It is possible that these opposing phenotypes may arise from different binding affinities of various forms of Tau (such as mutant, phosphorylated, or other conformers or isoforms of Tau) to synaptic proteins, such as, for example, the vesicular protein synaptogyrin-3 [92, 93, 276, 301, 503].

### The binding of tau to synaptic vesicles

In the pre-synaptic compartment, exocytosis of synaptic vesicles containing neurotransmitter is vital for the transmission of nerve impulses from the ‘pre-’ to ‘post-’ synaptic neuron via chemical synapses. To maintain a sustained release of neurotransmitters during periods of high synaptic activity, such as required for some forms of plasticity, a trafficking cycle occurs which can combine clathrin mediated endocytosis (CME) and the engagement of reserve pools of vesicles (for a review see [431]. The mediation of stages of this cycle are also highly Ca^2+^-dependent often due to Ca^2+^-dependent phosphorylation of synaptic proteins [94].

Tau is capable of mediating toxicity specifically via interactions with synaptic vesicle proteins and the prevention of vesicle release. Mutant (R406W, V337M or P301L) or phosphorylated Tau immobilises synaptic vesicles by preventing their release from F-actin. This reduced vesicle motility was hypothesised to occur through a mesh of immobilised vesicles formed by a crosslinking of the N-terminus of Tau with synaptogyrin-3 and its proline-rich and microtubule-binding domain binding to F-actin networks [136, 177, 194, 503]. The reduced vesicle mobility could be rescued by knock-down of synaptogyrin-3 or by depolymerisation of F-actin bundles [301, 503]. Deficits from this dysfunction, such as decreasing excitatory junction potential (EJP) amplitudes, are not seen from low frequency (0.2 Hz) stimulation that employ the recycling pool of vesicles for release, but only following high frequency (e.g. 10 Hz) stimulation requiring the reserve population of vesicles. Under high frequency stimulation, normal levels of release cannot be maintained and therefore result in impaired synaptic transmission. This work showed that this pathology only occurred with mutant FTLD or hyperphosphorylated Tau as opposed to wildtype Tau, which showed less synaptic colocalisation. However, it was also suggested that the formation of Tau multimers may also permit Tau to immobilise vesicles [503]. The above results are comparable to results on studies related to α-synuclein, which have shown that α-synuclein is equally capable to immobilise synaptic vesicles by aggregation [103, 461, 503]. As mentioned in Table 1, opposing effects of Tau have also been observed when measuring vesicle release probability. An increased release probability was shown to occur in 16 month-old mice expressing P30lL Tau in a subset of cells from the entorhinal cortex using a Tet-OFF system (rTgTauEC, [89, 353]. If mutations, phosphorylation or different conformations of Tau can alter its binding affinities with synaptic proteins, it could change the release probability of synaptic vesicles or influence the timing of other pathways required for the coordination of synaptic plasticity. Phosphorylation is known to alter binding properties and localisation of multiple other synaptic proteins including synapsin-1 [309], dynamin-1 [75], assembly of complexes to mediate Ca^2+^-dependent exocytosis [488], and post-synaptic AMPAR (α-amino-3-hydroxy-5-methyl-4-isoxazolepropionic acid receptor) and NMDAR (N-methyl-D-aspartate receptor) subunits [180, 298]. An interesting question arises from Tau’s ability to bind synaptic vesicles as to whether it is acting as a static tether and scaffolding protein, or has an active role in a mechanism at the synaptic compartment.

### Tau in the vesicle cycle of synaptic compartments

**Fig. 3:**
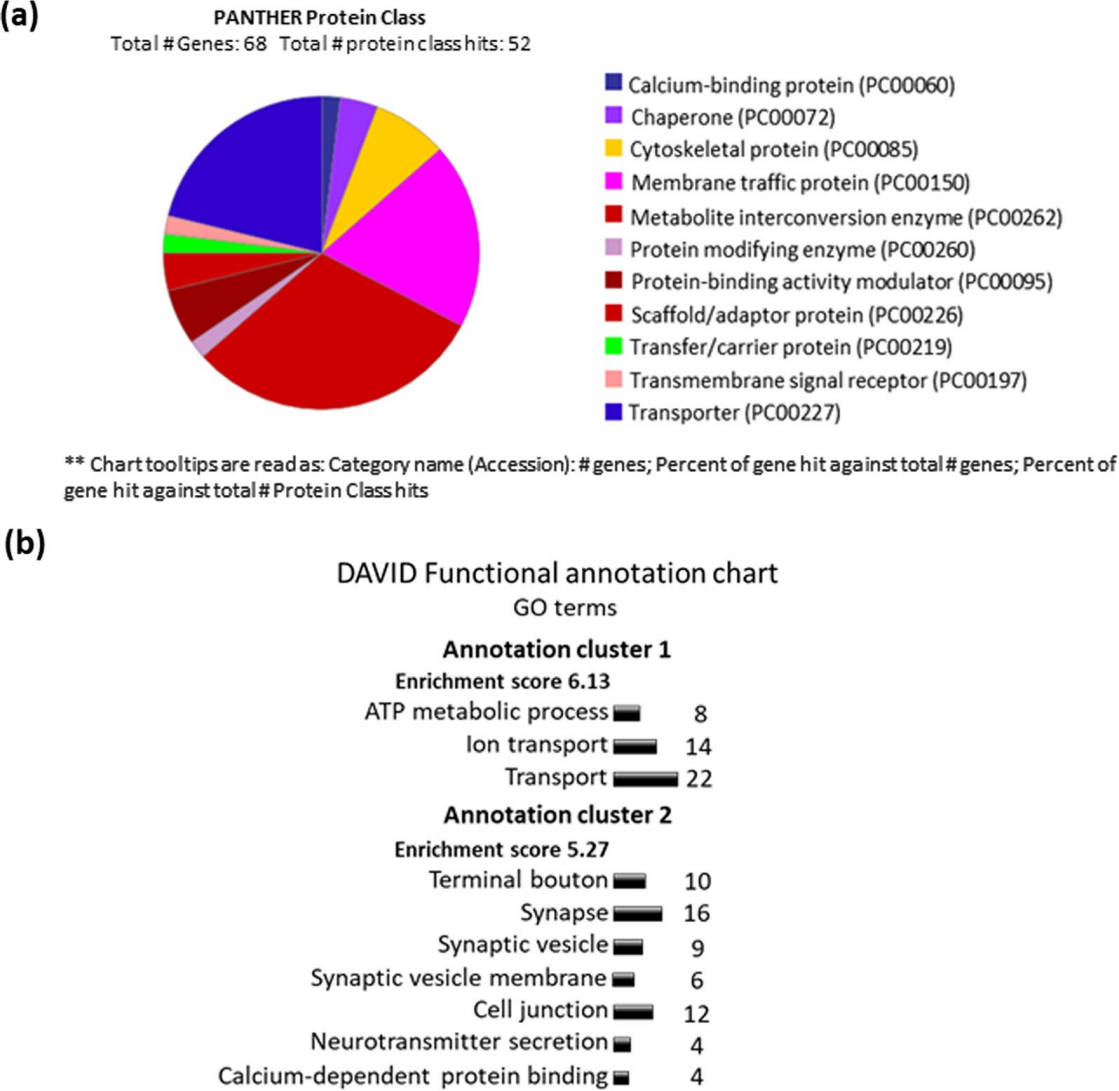
Bioinformatics analysis of Tau-interacting proteins suggests roles in scaffolding and transport with high enrichment at synapses and cell junctions. **a** Protein analysis through evolutionary relationships (PANTHER; [306] of proteins that bind Tau based on a co-immunoprecipitation study [276]. The proteins were classified according to their protein class. **b** Database for annotation, visualisation, and integrated discovery (DAVID GO annotation analysis [195, 196]. The two functional gene groups with the highest enrichment scores are shown for the 68 genes included in the annotation analysis

Bioinformatic analysis of Tau-interacting proteins based on co-immunoprecipitation studies by Liu et al. [276] show that many of these proteins are enriched in classes related to membrane trafficking and transportation, or metabolic activity (Fig. 3a). The functional annotation chart shows that these genes can be split into two functional groups with the highest enrichment scores (Fig. 3b). These clusters are related to metabolism and transport, and to synaptic processes. This suggests that Tau may have a role in membrane trafficking assisting in stabilising or transporting proteins. In the synapses, this could relate to processes such as CME and activity-dependent trafficking of membrane or proteins to support plasticity.

Clathrin mediated endocytosis is important for the internalisation of extracellular material and maintaining membrane homeostasis to balance exocytosis. CME requires the coordination of many endocytosis-related proteins for the formation of complexes at retrieved clathrin-coated pits on the membrane surface after calcium-dependent calcineurin is activated by neural activity [211, 478, 479]. At the pre-synaptic compartment, CME is the main mechanism through which the synaptic vesicle pool is replenished during physiological activity at the hippocampal synapse [162]. In neurons, it is estimated that ∼90% of all clathrin vesicles are involved in retrieval of synaptic vesicles [147]. At the post-synaptic compartment, CME regulates activity‐dependent endo- and exocytic trafficking of receptors [404]. CME is essential for activity-dependent AMPAR internalisation and LTD, and can therefore be upregulated by factors that induce synaptic depression such as NMDAR activation [12, 28, 114, 268, 288].

## Postulating synaptic roles of Tau based on binding studies

**Fig. 4:**
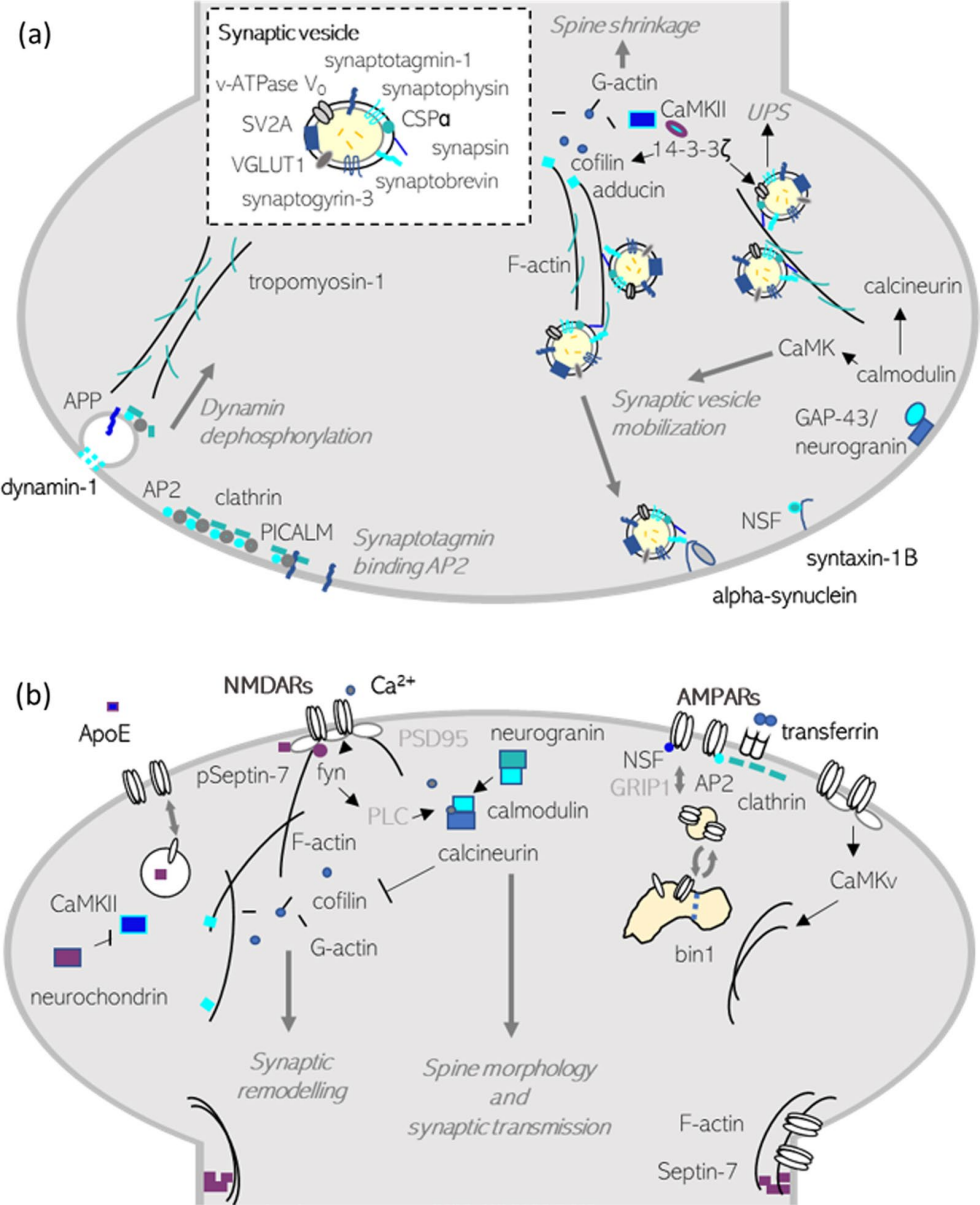
A schematic of possible roles of endogenous Tau in synaptic plasticity. The figure shows proteins that have been found to interact with Tau (though not necessarily functionally). Many of these proteins appear to map to clathrin-mediated endocytosis pathways that relate to synaptic vesicle trafficking in the pre-synaptic compartment (**a**) or receptor trafficking in the post-syanptic compartment (**b**) and are vital for synaptic transmission. Image based on data from [276]. Tau is not known to directly bind PICK1, GluA2, though there is an NMDA-dependent interaction of this complex with phospho-Tau [370]. These proteins are therefore added to the figure in order to highlight specific pathways that have been previously mentioned in the literature [434]

To help us to better interpret the spatial distribution of these proteins, Fig. 4 shows proteins that have functional roles inside synaptic compartments, and have been shown to be capable of binding to monomeric Tau by co-immunoprecipitation studies [276]. Many of these proteins appear to be related to clathrin-mediated endocytosis, and vesicle cycling pathways in synapses. These proteins have been mapped onto pathways that occur in the synaptic compartments to come up with a potential role of endogenous Tau which subsequently may become impaired during the progression of AD pathogenesis (Fig. 4). It is important to note that while the binding partners of Tau have been described, the functional roles of these interactions have not been experimentally proven to be directly linked to Tau and must therefore be seen as discussion points. GluA2 and AMPARs are not known to be direct binding partners of Tau but have been added as a potential candidates, as Tau may modulate the latter by indirect interactions with PICK1 (Protein interacting with C kinase) [370]. The full list of synaptic proteins that Tau is capable of binding to, are listed in Supplementary Table 1.

### Transferrin, AP2 and dynamin-1; possible role of tau in early stages of CME

Constitutive CME is required for plasma membrane protein and lipid turnover, endocytosis of activated growth-factor receptors, low-density lipoprotein and iron-saturated transferrin uptake [48, 79, 302]. Tau is capable of binding transferrin [276], which binds Fe^3+^ ions prior to clathrin-mediated uptake into cells via transferrin receptors. Transferrin receptor clustering is important for the initiation of clathrin coated pits for the earliest stages of CME to occur [275, 277]. Transferrin receptors are therefore also important for activity-dependent AMPAR internalisation that is dependent on CME and required for LTD as they recruit AP2 (adaptor protein 2). Tau is able to bind AP2, the protein responsible for clathrin pit formation [406, 448]. Loss of transferrin receptors also reduces LTP [264]. In the pre-synaptic compartment, following neuronal stimulation with KCl, Tau has been shown to relocate to the plasma membrane and to colocalise with, though not evidently bind, synaptic vesicle protein CSPɑ/DnaJC5 (Cysteine String Protein- ɑ) [503]. CSPɑ regulates endocytosis by binding dynamin-1, another protein capable of binding Tau that is involved in activity-dependent CME of synaptic vesicles through vesicle scission [126, 127, 386, 478, 479, 497].

PICK1 makes NMDAR-dependent interactions with endocytic proteins AP2 and dynamin. Following NMDAR stimulation, PICK1–AP2 interactions cluster AMPARs at endocytic zones, and PICK1 can polymerize dynamin-1 to undergo AMPAR endocytosis [130]. The preference of different Tau isoforms to bind to the proteins shown in Liu et al. [276] can be seen in Supplementary Table 1 [237].

### Possible role of Tau in SNARE complex formation and exocytosis: syntaxin-1, synaptobrevin, NSF

CSPɑ is also essential for the high Ca^2+^-sensitivity of exocytosis as it mediates the release of anchored synaptic vesicles by formation of the Ca^2+^-sensitive SNARE complex (Fig. 4a; [65]. Like Tau, CSPɑ can bind synaptotagmin, and proteins involved in the SNARE complex including syntaxin-1 and synaptobrevin [121, 330, 400, 477]. SNARE complex assembly requires SNAP-25 (Soluble NSF Attachment Protein) and syntaxin-1 to bind to synaptobrevin to exert sufficient force for membrane fusion to occur and to release the vesicle contents into the synaptic cleft [417]. This assembly is disrupted in CSPɑ-deficient mice [403]. CSP-KO in itself can induce neurodegeneration, and in *Drosophila* prevents the release of neurotransmitters and causes early death [386, 451, 507].as CSPɑ is thought to induce the required structural conformation of SNAP-25 and prevent its degradation by the ubiquitin proteasome system (UPS), which degrades excess or damaged proteins [403] Tau is also able to bind the protein required for SNARE disassembly, NSF (N-ethylmaleimide sensitive fusion protein) (Fig. 4, [276, 417]. The role that CSP may play in Tau-mediated neurodegeneration is being questioned following the finding that CSP expression is downregulated in tauopathy models at timepoints that correspond to impaired synaptic function. In these models, CSPɑ was also found to be neuroprotective, whereby increased expression reduced neuronal loss [445].

CSPɑ/DnaJC5 bound to Hsc70 releases Tau from synapses in what is believed to be a physiological, activity-dependent mechanism [131]. It will be interesting to determine whether CSPɑ loss in tauopathies also reduces activity-dependent Tau release [355], and whether this leads to a clear phenotype. Other DnaJ proteins complex with Hsc70 for disaggregation [141, 331] or degradation [207] of aggregated proteins. Aggregated proteins can directly block CME through competition for Hsc70 [492]. It has been suggested that CSP may act as a chaperone to allow continuous and long-term use of proteins in the synaptic vesicle cycle [125].

### ɑ-synuclein and 14–3-3ζ

The fatal phenotype caused by CSPɑ, that prevents vesicle release, is rescued by overexpression of ɑ-synuclein [66]. ɑ-synuclein is another pre-synaptic protein thought to have a role in the synaptic vesicle cycle including endocytosis [454], reclustering [327], and mobility [398, 461] but is found in Lewy body aggregates seen in Parkinson’s disease (for an overview on ɑ-synuclein induced synaptopathy see [45]. Tau can bind to both ɑ-synuclein and β-synuclein [204, 276]. Co-morbid ɑ-synuclein or Lewy-related pathology occur in more than 50% of AD brains, and ɑ-synuclein and Tau have synergistic effects on each other’s aggregation [145, 169, 272]. ɑ-synuclein and Tau are thought to form a membrane-bound complex with the actin cytoskeleton. Destabilisation of the cytoskeleton or the A30P ɑ-synuclein mutation linked to early-onset Parkinson’s disease reduces the formation of this complex [118, 243, 340]. ɑ-synuclein can also induce Tau phosphorylation at serine 262 to cause unbinding from actin and microtubules, and has been shown to be essential for Aβ42-induced Tau toxicity [56, 198, 204].

ɑ-synuclein shares functional homology with the highly conserved regulatory 14–3–3 proteins that are able to bind both ɑ-synuclein and Tau [276, 337]. Tau has also been found capable of binding to the zeta isoform of 14–3-3 proteins (14–3-3ζ), which are enriched in the hippocampus, especially in synapses, and thought to be involved in learning and memory pathways [27, 88, 276, 293, 412, 466]. Overexpression of 14–3-3ζ increases Tau phosphorylation at serine 262, actin unbinding, and depolymerisation of microtubules through the same pathway as ɑ-synuclein, and consequently leads to the degradation of synaptophysin by the UPS [204, 364]. 14–3-3 is also capable of phosphorylation-dependent binding to CSPɑ/DnaJC5 [359], and plays a role in priming exocytosis and enhancing vesicle release through structural rearrangements of the actin cytoskeleton [64, 382]. Alternatively, 14–3-3ζ can coordinate, together with other DnaJ-Hsc70 complexes, the resolubilization of heat-aggregated proteins [486].

### V-ATPase

14–3–3ζ has an ATPase activity and helps to regulate vacuolar-type H^+^-ATPase (V-ATPase) activity [1, 5, 367]. Tau is able to bind to V-ATPase subunit A, required for the acidification of intracellular compartments for maintaining synaptic vesicle proton gradients, protein sorting, and receptor-mediated endocytosis. Loss of this protein impairs late stage exocytosis of synaptic vesicles. Mutations in V-ATPase subunits can cause epilepsy and parkinsonism [168, 240], cognitive impairment, and neurodegeneration [110].

### Cytoskeletal plasticity: cofilin, troponomyosins and septin 7

Another interaction 14–3–3ζ can mediate, in concert with CaMKII (Ca^2+^/calmodulin-dependent protein kinase II) and in opposition with Ca^2+^/calmodulin-activated phosphatase calcineurin, is the dephosphorylation and activation of the actin organising protein cofilin [229, 500]. Tau can directly bind cofilin, CaMKII and calcineurin [273, 276, 490]. Cofilin can compete with Tau for tubulin binding which has been suggested to cause microtubule instability and promote tauopathies through increasing free Tau available for fibril formation [473]. The ability of both cofilin and Tau to bind to tubulin and actin suggests that they coordinate cytoskeletal plasticity pathways. Ca^2+^ entry through NMDARs can cause the indirect dephosphorylation and activation of cofilin through calcineurin. This causes cofilin to enter synaptic compartments and depolymerise F-actin to cause spine shrinkage. Overactivation of this pathway during stress can cause the transient cofilin-actin rod response that bundles actin and releases ATP. This response can occur in Alzheimer’s disease causing long-term F-actin bundles in axons and neurites [19, 287, 323]. In Tau-P301S mice, activated cofilin is also required for tauopathy, reduced synaptic integrity (as shown by depleted drebrin and synaptophysin, and LTP deficits; these deficits were rescued in mice having a 50% reduction in cofilin concentrations [473]. This reduction in cofilin also rescued loss of synaptic proteins and impairment to LTP in APP/PS1 mice [472]. Cofilin is important for spine dynamics during LTP and LTD, as well as for AMPAR trafficking, for example, following chemical induction of LTP, activated cofilin results in increased surface AMPARs [69, 165, 504].

Tropomyosin is another actin-associated protein that stabilises F-actin and that Tau is capable of binding to in vitro [276]. Tropomyosin recruits cofilins to F-actin and they help to determine the structure of pre-synaptic F-actin and the stiffness of the pre-synaptic membrane [31, 51, 430].

Septins are seen as the fourth filament protein in neurons alongside actin, tubulin, and neurofilaments. They help regulate synaptic vesicle trafficking and neurotransmitter release [296], and septin 7 interacts with the exocyst complex [193]. Septins can bind with actin during various stages of CME and endosomal sorting, which is required for the maintenance of mature synapses, and synaptic plasticity such that septin 7 expression is up-regulated during spatial memory formation [117, 457]., which is impaired in AD. In dendrites, septin 7 binds to the membrane of hippocampal neurons to regulate dendrite branching and spine morphology but it can also prevent the lateral diffusion of membrane proteins out of spines [122, 481]. Following phosphorylation, septin 7 stabilises post-synaptic density (PSD) protein PSD95 during spine maturation [483]. Several septins are also found in NFTs [232].

### *Ca*^*2*+^*-dependent interactions: calcineurin, GAP-43/neuromodulin, neurogranin, neurochondrin, calmodulin, CaMKII, and CaMKv*

Further to calcineurin activating cofilin, Tau is capable of binding calcineurin, GAP-43/neuromodulin, neurogranin, neurochondrin, calmodulin, CaMKII, and CaMKv (calmodulin kinase-like vesicle-associated), which have been shown to interact at synapses [36, 87, 266, 276, 349, 415, 490]. Calcineurin has long been known to regulate activity-dependent cytoskeletal remodelling; it is able to dephosphorylate Tau to polymerise and stabilise microtubules opposite to CaMKII [159]. Overexpression of Tau or Aβ oligomers have been shown to increase the activation of calcineurin [384, 487]. Calcineurin inhibition however, can rescue spine density and plasticity deficits in AD model mice [63, 366, 384]. Calcineurin can regulate the available concentration of calmodulin at the pre-synaptic compartment through dephosphorylation of GAP-43, which also causes actin capping [40, 178, 256]. GAP-43 can cause presynaptic membrane changes, and is thereby involved in neurotransmitter release, endocytosis [329], synaptic vesicle recycling, LTP, and spatial memory formation [96]. GAP-43 has a high affinity for calmodulin at low Ca^2+^ concentrations, sequestering calmodulin at the cell membrane until Ca^2+^ influx occurs [11]. Tau helps maintain this cytoplasmic concentration of calmodulin available through Ca^2+^-dependent binding to prevent it from entering the nucleus [24, 415]. Calmodulin is important for the activation of CaM-dependent kinases. CaMKII has been shown to phosphorylate Tau at sites including serine 262 that promotes microtubule unbinding [411] and is thought to be involved in the formation of fibrillar Tau [179, 490]. CaMKv is upregulated following AMPAR activity to cause actin rearrangement, and is said to be the ‘convergence point for the transduction of Ca^2+^ signals to the neuronal cytoskeleton’ [266]. The calmodulin/calcineurin pathway has also been show to directly modulate endocytosis through dephosphorylation of endocytic proteins including dynamin-1 [74, 75, 433].

### MBP

Myelin basic protein (MBP) is the second most abundant protein in the central nervous system. Like Tau, MBP is an intrinsically disordered protein with multiple isoforms, and can be differentially phosphorylated suggesting that it has a role in neuronal signalling. Like Tau, MBP is also capable of binding tropomyosin, actin, microtubules, calmodulin, and clathrin [39, 106, 314, 358]. As MBP can act as a clathrin adaptor protein, it has been suggested that it may form a bridge between clathrin-coated vesicles and microtubules [358]. MBP can also polymerise, bundle and crosslink actin filaments and microtubules, and act as a tether for SH3-domain proteins to lipid membranes (such as for the SH3-domain of Fyn-kinase) [38, 39, 188]. Although Tau is able to bind MBP, Tau and MBP appear to have analogous roles in neurons versus oligodendrocytes, respectively. MBP is important for formation and stabilisation of the cytoskeleton in oligodendrocytes [113, 138]. MBP forms prion-like aggregates, in parallel to the accumulation of insoluble and phosphorylated Tau, and can occur due to reduced cholesterol levels and other lipids that cause MBP-membrane unbinding [133, 285, 470]. Like Tau, MBP can aggregate due to polyanionic factors such as lipids or lysosomal glycosaminoglycan (GAG) proteins [146].

### Fyn

A small amount of Tau is found in dendrites and spines under physiological conditions and can be phosphorylated following NMDAR activation [317]. Phospho-Tau can facilitate the interaction of Fyn kinase, PSD95 and NMDARs to stabilise their position in the postsynaptic density [160, 201, 255, 313, 317]. The interaction of Tau and Fyn was previously predicted to cause the translocation of Tau to cholesterol-rich lipid rafts to act as a signalling protein [259]. The entry of Tau into synapses is also thought to regulate the activity-dependent transportation of synaptic proteins, including Fyn kinase, GluA1 and PSD95 [225, 317]. Transport of PSD proteins is required to allow synaptic plasticity [115, 425]. At the postsynaptic density, this complex has been implicated as the mechanism of Aβ -induced excitotoxicity caused during AD pathology through overactivation of NMDARs and phosphorylation of Tau at tyrosine 18 (Y18) [313, 389]. Y18 is also associated with the formation of insoluble Tau aggregates [47, 258]. Tau knockout has been shown to be neuroprotective by ameliorating Aβ-induced excitotoxicity, by causing the exclusion of Fyn from the post-synaptic compartment and by destabilisation of PSD-95 [200, 201]. Another recent paper has shown that post-synaptic FTLD-mutant Tau causes aberrant Fyn nanoclustering in hippocampal dendritic spines [338]. Fyn knockout causes impairments to LTP and spatial learning in mice, this is specific to Fyn as opposed to other nonreceptor tyrosine kinases [163]. Other than being linked with excitotoxicity, the phosphorylation of Tau at serine 396 has also been shown to be required for hippocampal LTD [370]. Although the exact mechanism was not described, it was shown that Tau is necessary for an activity-dependent molecular interaction between GluA2 and PICK1, both of which are required for the internalisation or stabilisation of intracellular pools of AMPARs [170, 280, 370, 443]. GluA2 subunits in AMPARs render them Ca^2+^ impermeable [52, 186, 418]. The GluA2 subunit can also bind NSF and AP2 for stabilisation versus internalisation [99, 262, 332, 336, 419, 480]. As NSF and AP2 binding sites on GluA2 overlap, they are thought to elicit different functions, which may explain the complexities of AMPAR trafficking [262]. As well as its involvement in the GluA2-PICK1 interaction, Tau has also been shown capable of binding to NSF and AP2 by co-immunoprecipitation studies [276]. The function of this binding may be related to NMDA-induced trafficking of AMPARs from synapses, whereby Tau deficiency results in reduced GluA2 subunits in the postsynaptic density during chemical LTD [434]. GluA2 also regulates metabotropic glutamate receptor-dependent LTD (mGluR-LTD) through a pathway involving cofilin-mediated actin reorganisation [506].

**Fig. 5:**
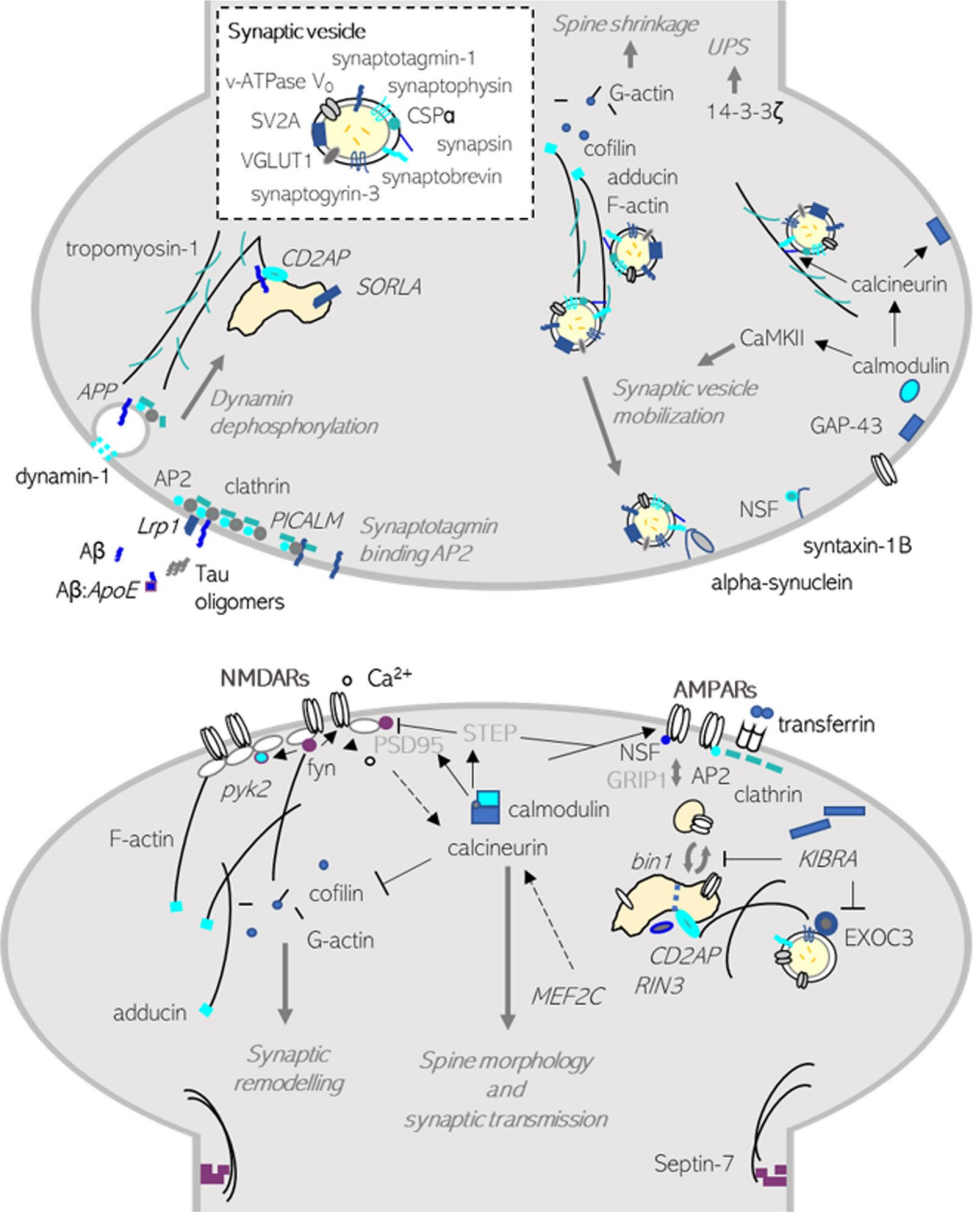
The genetic risk factors for Alzheimer’s disease are involved in synaptic plasticity. Proteins that have genetic links to AD (italicised) are mapped into the pathways described for proteins that are capable of binding to Tau. Proteins used to demonstrate the pathway but that do not bind directly to Tau (purple) or are known genetic risk factors (italicised) are also shown

Tau can bind several proteins, interact with, or is directly involved in various stages of CME and synaptic trafficking, the proteins of which are also genetic risk factors for AD. These include the top three genetic risk factors, *APOE* [428], *BIN1* [67, 217] and *PICALM* [174, 248]. Similarly, many genetic risk factors for AD have been linked to CME though these proteins may not be known to directly bind to Tau, and will therefore be discussed in more detail (Fig. 5).

## Possible roles of synaptic Tau based on interactions with proteins identified as genetic risk factors in Alzheimer’s disease

Another hint that CME may be closely associated with AD pathology comes from genetic mutations that can lead to late onset Alzheimer’s disease (LOAD) including *PICALM* [174, 248], *EXOC3L2 (*Exocyst complex component 3-like 2; [335, 401], *BIN1* [217], MEF2 (myocyte enhancer factor-2; [61], KIBRA [83], RIN3 (Ras and Rab Interactor 3; [217], and Sorla [379]. Figure 5 illustrates how these genetic risk factor proteins may further interact with pathways involved in synaptic plasticity, or with the proteins previously discussed in Fig. 4.

### PICALM

Picalm is an adaptor protein required for clathrin-mediated endocytosis by directly binding to clathrin, phosphatidylinositol, and AP2 to help form clathrin-coated pits on the cell membrane [109, 305, 441]*.* Overexpression or degradation of Picalm blocks endocytosis, and has also been related to the extent of Tau pathology [7, 8, 228, 385, 441]. Picalm may be involved in CME-mediated uptake of Tau species and is also found bound to fibrillar Tau [6, 475]. Increased concentrations of calpain-cleaved Picalm are found in the AD brain, which has been hypothesised to impair endocytic function in AD [6].

### APP

Genetic mutations in the Amyloid precursor protein (APP), from which the fragment Amyloid-beta (Aβ_1-42_) arises, can be a direct cause of AD [149]. APP is only partially processed at the cell surface but requires CME for further processing and its synaptic activity-dependent release [73, 239, 247, 455]. APP has functional roles in regulating spine density whereby overexpression or knockdown of APP causes an increased or reduced number of spines, respectively [261]. Aβ can cause dysregulation of intracellular Ca^2+^ concentrations [13, 244], and synaptic depression, thought to occur by aberrant trafficking and excessive removal of AMPARs [166, 191]. This could induce LTD through NMDA receptor- and metabotropic glutamate receptor-mediated signaling [166, 281]. APP has been shown to bind to Tau [199] without any known role, a functional link between these proteins however occurs via Fyn kinase. As previously discussed, both Tau and Aβ have been related to neuronal excitability, however memory deficits, excitotoxic seizures, and seizure-induced premature mortality of mice overexpressing the Swedish mutation of APP (APP_Swe_) was reduced when nonreceptor tyrosine kinase, Fyn, was unable to enter post-synaptic compartments due to the expression of a truncated form of Tau [200]. For review on Aβ and trafficking see Perdigão et al. [346]. For reviews on synaptic mechanisms of Tau pathology see [107, 346].

### BIN1, RIN3 and CD2AP

BIN1 is involved in endocytosis by binding to clathrin and AP2 during early endosome formation, and in the exocytosis of vesicles from recycling endosomes to the cell surface [57, 397]. Loss of BIN1 causes impaired presynaptic vesicle distribution and release, reduced synaptic density due to membrane trafficking, and an altered presynaptic protein clustering [381]. In the postsynaptic compartment, this loss also causes reduced surface expression of the GluA1 AMPAR subunit in the post-synaptic compartment and altered AMPAR-mediated synaptic transmission [397]. Overall, BIN1 knockout leads to deficits in synaptic transmission, and impaired spatial memory consolidation at the behavioural level [381]. Tau reduction has also been shown to reduce network hyperexcitability mediated by BIN1-interactions with L-type voltage-gated calcium channels (LVGCCs) [458]. BIN1 can rearrange the actin cytoskeleton and stabilise Tau-induced actin bundles [108]. BIN1 and Tau colocalise at the actin cytoskeleton [421] as has also be shown for BIN1 with the genetic risk factor protein RIN3 [217, 383] to mediate receptor-induced endocytosis and transport of vesicles from the plasma membranes to early endosomes [212]. BIN1 and RIN3 are both able to bind CD2AP (CD2 associated protein), a protein found in cases of sporadic AD [185, 324, 328, 383, 439]. This interaction has been related to regulating cholesterol, which has been linked to AD through genetic risk factors involved with cholesterol homeostasis [62, 295], and increased incidence of AD in hypercholesterolemia [295, 341, 407]. In *Drosophila*, loss of the CD2AP ortholog, *cindr,* causes a combination of endocytic and exocytic synaptic defects including impairments of synaptic vesicle recycling and release [334, 408] and enhanced Tau-induced neurodegeneration [408]. Loss of *cindr* causes defects in endocytosis as shown by depression of synaptic responses during high-frequency stimulation, as mutants are unable to sustain synaptic vesicle release [30, 173, 237]. It is thought that impairment to synaptic vesicle endocytosis may be through the ability of CD2AP to link the binding of clathrin and actin via cortactin [282, 334, 499]. CD2AP also binds actin, whereby its loss of function stabilises F-actin [210, 469]. Exocytosis of vesicles is also affected by cindr having a role in presynaptic Ca^2+^ homeostasis*.* This is thought to occur through binding 14–3-3ζ to regulate the UPS for activity-dependent proteostasis to control the degradation of proteins involved with plasticity [334]. It is possible that Tau influences this pathway as 14–3-3 can increase Tau aggregation and co-immunoprecipitation studies have shown that Tau can bind to 14–3-3ζ, though the functional relevance of this remains to be determined [182, 276]. The UPS has an important role in endocytosis, protein trafficking, the size of post-synaptic potentials and the formation of long-term memory [202, 422, 502]. Alpha-synuclein, synaptophysin, syntaxin1, SNAP-25, synapsin1, GluA2, PSD95 and plasma membrane calcium ATPase (PMCA) have been identified as pre-synaptic targets for the UPS [29, 71, 78, 283, 334, 344, 403, 471]. AMPAR subunits are targets for degradation by the UPS for LTD induction following uptake by CME [115]. This occurs through ubiquitination of PSD-95 which otherwise acts as tether for AMPARs and shields them from degradation [78]. Burbea et al. (2002) hypothesize that there is an intricate link between ubiquitination, clathrin-mediated endocytosis and UPS degradation, suggesting activity-dependent ubiquitin-conjugation of AMPARs to influence AMPARs at synapses [344]. Unregulated deubiquitination of synaptic proteins can also result in synaptic overgrowth and blocked release of synaptic vesicles [102]. Like Picalm, Bin1 and CD2AP depletion can cause impaired vesicle recycling or release and result in an accumulation of Aβ and other proteins inside of early endosomes [450]. This may also influence the aggregation of Tau, which is promoted at low pH inside of endolysosomal compartments [307].

### MEF2C

MEF2C is a transcription factor that regulates hippocampal-dependent learning and memory through the control of dendritic spine density, miniature excitatory postsynaptic currents (mEPSCs) frequency, probability of vesicle release, and activity-dependent AMPAR trafficking through its presence in the pre- and post- synaptic compartments [20, 77, 221, 368]. *MEF2C* is therefore important for activity-dependent refinement of synaptic connectivity in homeostatic plasticity [20].

### EXOC3L2

*EXOC3L2* is a component of the exocyst, involved in the exocytosis of vesicles containing hormones, extracellular components, membrane lipids, and for the regulation of the readily releasable pool of synaptic vesicles via the binding of NSF and SNARE proteins including syntaxin1 [189, 354]. The activity-dependent addition of membrane to the synapse via the exocyst is required for synaptic plasticity [442]. The exocyst interacts with postsynaptic density proteins to regulate NMDAR and AMPAR trafficking and exocytosis at the postsynaptic membrane [143, 390]. Overall, the exocyst acts as an integrator between the secretory pathway and cytoskeleton, including septins, actin, and microtubules, to localise vesicles to release sites [192, 436, 456].

### KIBRA

KIBRA is enriched in brain regions involved with memory such as the hippocampus and cortex, where it is found in the perinuclear and somatodendritic regions of neurons, particularly at postsynaptic densities. KIBRA acts as a postsynaptic scaffold protein that connects the cytoskeleton with signalling molecules [208]*.* KIBRA is capable of binding activity-dependent AMPAR regulators including NSF, PSD-95, PICK1, GluA1, GluA2 and GRIP1 (Glutamate receptor-interacting protein 1), and is involved in AMPAR recycling, through its ability to bind with the exocyst complex [286, 380]. By binding the exocyst, KIBRA can direct PKMζ (Protein kinase Mzeta), a brain-specific variant of PKCzeta that plays important roles in memory formation, to required locations which is why it has been hypothesised to be a ‘synaptic tagging’ protein [489]. PKMζ is necessary and sufficient for enhanced synaptic transmission during LTP maintenance and acts by increasing the number of postsynaptic AMPARs [270, 271]. PKMζ, but not other PKC isoforms, has been found in NFTs in brain regions specifically involved with memory loss in AD, whereas they are not found in NFTs of control brains without memory impairment [85]. As KIBRA is involved in AMPAR recycling, knockdown of KIBRA results in an increase of AMPAR recycling following NMDAR internalisation [286]. This mechanism is impaired by Tau in AD. Acetylated forms of Tau seen in AD brains (K274 and K281) promote memory loss by preventing the recruitment of KIBRA into post-synaptic compartments, causing impaired activity-dependent postsynaptic actin remodelling and AMPAR insertion [447].

### PTK2B

*PTK2B* encodes Pyk2 (proline-rich tyrosine kinase 2), a susceptibility factor for AD [248]. Fyn kinase can activate Pyk2, which then binds and phosphorylates Tau [59, 263, 361]. Pyk2 interacts with NMDARs, dependent upon binding PSD95, to phosphorylate NR2 subunits and increase receptor conductance during the induction of LTP [238, 399, 463]. Pyk2 binding to PSD95 is activity-dependent as it requires activation of calmodulin by Ca^2+^ (Fig. 5, [25]. Pyk2 is fundamental to synaptic dysfunction triggered by Aβ as mice lacking Pyk2 were protected from synapse loss and memory impairment [388].

### LDLR and ApolipoproteinE

The LDLR (low density lipoprotein receptor) has been linked to AD both through direct mutations and through interaction with ApoE (apolipoprotein E), the highest genetic risk factor for LOAD [81]. LDLR is involved in cholesterol uptake via CME. Cholesterol is essential for the maintenance of mature synapses to increase the number of synaptic vesicles and release sites, and overall release efficacy [148, 410]. Another low density lipoprotein receptor found at the postsynaptic density, LRP1, has been found to be the major receptor for monomeric or oligomeric Tau uptake, and can also cause age-dependent synaptic loss and neurodegeneration in a knockout mouse model [80, 275, 277, 300, 369]. Oligomeric hyperphosphorylated Tau can bind and be released from cells by HSPGs prior to binding LRP1 [80, 218, 304]. Apolipoprotein E deficient mice show heparan sulfate-enhanced low density lipoprotein (LDL) aggregates that are taken up by LRP1, causing cholesteryl ester accumulation in macrophages and production of atherosclerotic plaques [267, 289]. LRP1 is also responsible for the endocytosis and degradation of Aβ, or Aβ-ApoE complex, whereby amyloid pathology is enhanced by the APOE4 allele, dependent upon LRP1 uptake [90, 214, 437]. Alongside this, LRP1 is involved in the endocytosis of APP, which is required for its processing of Aβ peptides [234].

The ApoE type 4 allele, the highest genetic risk factor for AD [81], causes impaired vesicle cycling to the cell surface resulting in intracellular cholesterol accumulation [181, 428]. This impaired cycling also traps AMPARs and NMDARs clustered with ApoE receptors inside of endocytic vesicles, causing synaptic dysfunction [70]. Aβ is capable of regulating the surface expression versus endocytosis of NMDARs, potentially through disruption to their binding with PSD95 [377, 414]. Impaired cycling by ApoE4 is thought to exacerbate Aβ induced endocytosis of AMPA and NMDARs, and ApoE4 knock-in mice show increased sensitivity to the blockade of LTP by oligomeric Aβ and by failing to restore Reelin signalling [70, 414, 449]. In a pathway suggested by Durakoglugil et al. [112], Aβ competes against nonreceptor tyrosine kinase signalling, predominantly by Fyn kinase, over activation or antagonism of the Reelin pathway. In the absence of phosphorylation, the microtubule binding region of Tau is capable of binding ApoE3 but not ApoE4, [429]. ApoE4 also increases Tau-mediated neurodegeneration as compared with other alleles or knockout of ApoE, which is neuroprotective [405].

There are many genetic risk factors linked to AD that involve proteins required for CME, vesicle cycling or exocytosis. Alongside PICALM, BIN1, and Apolipoprotein E, which directly bind Tau, a number of proteins link genetic risk factor proteins with synaptic proteins capable of binding Tau and which are linked to vesicle cycling pathways [67, 276, 421]. Such proteins, recurring through this review, may include AMPARs, 14–3-3ζ, NSF, PSD95, Fyn kinase and clathrin itself. Many of the proteins which are able to bind Tau, or are related to LOAD and familial AD, also bind to the actin cytoskeleton and may therefore act as a linker between structural and signalling roles required for synaptic plasticity and memory mechanisms.

## Tau as a linker between CME, vesicle trafficking, and the cytoskeleton

Tau is able to simultaneously bind actin and microtubules, and induce the polymerisation of actin along microtubule tracks [116]. As Tau has been linked with physiological and pathological actin structures, it is worth discussing how these cytoskeletal arrangements may link with CME and previously discussed synaptic trafficking mechanisms.

CME is intricately linked to actin dynamics though the exact stage, location and function of these associations in mammalian cells have been strongly debated. It appears that actin is involved with the invagination of membrane and late stages of CME [139]. Actin has been suggested to play structural role and mechanical roles in exerting force during scission and constriction steps required for vesicle endo- and exocytosis [203, 363, 413]*.* Actin is the main cytoskeletal component of synaptic compartments and spines, and is thought to facilitate the cytoarchitectural changes required for synaptic plasticity [60, 104, 299].

Actin has also been suggested to have an active role in the segregation of vesicle populations to determine their retention or release at the membrane surface [72]. In the pre-synaptic compartment, loss of F-actin integrity has shown impairment to synaptic vesicle release or recycling in multiple studies [409, 459, 501]. This greatly reduces the number of synaptic vesicles in the stimulated condition due to the inability to retrieve vesicles from the plasma membrane [409]. Stabilisation of F-actin by phalloidin also prevents neurotransmitter release [32, 350]. Pre-synaptically, the bundling and stabilisation of actin by phalloidin are far more dramatic following the induction of action potentials, which cause the assembly of filamentous actin fibres, tethered with vesicles, from the endocytic zone to the periphery of the vesicle pool [409]. This may be similar to the effects seen when Tau is shown to cause increased resistance to depolymerising drugs by directly stabilising actin [136]. Decreased actin dynamics through actin bundling has previously been associated with senescence, whereas knockdown of the actin bundling protein SM22/transgelin increases longevity [161].

F-actin also determines the mobility of receptors between the cell surface and the cytoplasm [2]. In the post-synaptic compartment, F-actin stabilises receptors in dendritic spines, whereby its disruption decreases the number of NMDAR and AMPAR clusters. In hippocampal neurons, post-synaptic actin depolymerisation causes AMPAR endocytosis, similar to that induced by glutamate [2]. By contrast, stabilisation of F-actin can inhibit AMPAR internalisation [2]. The specific linker proteins that allow F-actin to facilitate these functions are not fully known. Much is still poorly understood about how CME and its role in vesicle cycling and plasticity links with actin and its mechanical and structural roles within the synaptic compartments. A number of proteins are responsible for actin dynamics, including the previously discussed proteins tropomyosin, cofilin, and adducin. Alongside the better known pathology of Aβ plaques and neurofibrillary tangles seen in AD, actin-depolymerizing factor (ADF)/cofilin-actin rods can also occur [310] which may be precursors to Hirano bodies, actin-rich inclusions that contain tropomyosin, Tau, and cofilin, among other proteins [140, 146].

As Tau is capable of binding the filament proteins septin7, tubulin and actin, Tau may act like a Velcro that reversibly positions structures into place for signalling pathways and to restructure proteins depending on the levels of synaptic activity. This role may balance the level of proteins available for function versus their degradation through cleavage by calpain and the UPS. This has already been discussed for the protein synapsin1, however numerous other proteins including actin, cortactin, NMDAR and AMPAR subunits, PSD95, SNAP-25, GAP-43, and GRIP are either targeted to scaffolding proteins such as PSD95 for stabilisation, or else marked by cleavage or ubiquitination for degradation [9, 33, 78, 348, 357, 457, 474, 494]. Reduced post-synaptic glutamate receptor localisation was proposed to be due to a depletion of PSD95 in the post-synaptic compartment, resulting in smaller postsynaptic densities following a reduction or mutation of Tau [325, 465]. The UPS is only responsible for the local degradation of a subset of synaptic proteins, and its function is regulated by synaptic activity or neural growth factors (NGF) to adjust the concentration of proteins important for synaptic function. This activity-dependent or NGF-dependent UPS function can thereby feedback to regulate neurotransmitter release and synapse elimination [206, 254, 372, 422].

## The role of Tau trafficking, its link to endocytosis, cholesterol and the cytoskeleton

A question that emerges from the above findings is whether the presence of Tau at different locations in the cells are due to internal Tau translocations or due to Tau being released into the extracellular space and its re-uptake by neighbouring neurons. There have been many reports on Tau trafficking and its uptake mechanism. It is thus interesting to note that several endocytosis-related pathways are involved in Tau trafficking and thus may explain why Tau pathology is linked to these different pathways (for a review see [50]. Furthermore, there are several studies emerging highlighting the role of the extracellular and intravesicular environment on protein misfolding involving high sodium, zinc, and calcium ion concentrations and solvents [319, 426, 426], low pH [307], presence of glucosaminoglycans [227, 321] to name a few. Recent studies [80, 369] also highlights another potentially important factor, namely cholesterol. Cholesterol has long been seen as a player in AD and many other neurodegenerative diseases, such as PD, Nieman Pick’s disease Type C (NPC) and ALS (for a review see [294]. The increased membrane-associated cholesterol concentration in the brains of patients with sporadic AD correlates with the disease severity [86, 291, 482]. In ageing neurons cholesterol is mainly taken up by endocytosis, as opposed to cell-autonomous cholesterol synthesis [137], and thus extracellular Tau and cholesterol may end up in the same endosomal compartment. Increased accumulation of Tau and cholesterol in endosomes may interfere with the WASH complex, similar to what has been observed in VPS35 (vacuolar protein sorting 35) related to PD [495], and thus affect the actin skeleton and endosome-lysosome networks [97, 154, 155]. Impaired cholesterol transport would not only reduce cholesterol being supplied to other organelles such as the mitochondria and the plasma membrane and lipid rafts, but also reduce the number of synaptic vesicles being formed.

## Conclusion

In AD, Tau is commonly discussed with regards to pre-synaptic [301, 503] versus post-synaptic [200] pathology, though little emphasis is put on mechanisms that may target common plasticity pathways such as synaptic protein and lipid trafficking, and vesicle cycling. In this review paper, we have highlighted synaptic proteins that Tau is capable of binding to, or genetic risk factor proteins, and mapped these to pathways that relate to plasticity mechanisms that would directly link Tau with impaired memory, a primary symptom of AD [23]. It is also important to note that changes to memory mechanisms occur even as a result of healthy ageing. In general, it has been shown that in older animals LTP is less robust and requires stronger input whereas LTD is enhanced [22, 318, 333, 446]. AD pathology may further hijack these mechanisms leading to symptoms of dementia.

A small amount of Tau has been detected at synapses under physiological conditions. Due to the activity-dependence of Tau translocation to synapses, it has been hypothesised that Tau may have a supporting structural role during development and plasticity [190, 132, 317, 392, 434, 438]. Tau has been suggested to coordinate microtubule and actin dynamics to allow structural alterations during activity, as Tau binds F-actin with a physiological function [136, 177, 493]. Tau has been found to be capable of binding to a number of proteins with roles associated with clathrin-mediated endocytosis (Fig. 4). It is plausible that Tau acts as either a tethering protein between vesicles, similarly to synapsin, at least during pathological conditions [301]. This binding may occur with microtubules, septin, actin-mediated mechanisms, or HSPG extracellular matrix for either supplying, stabilising, or transporting components required for plasticity. Phosphorylation-dependent mechanisms that change protein interactions and synaptic scaffolding may become impaired in pathways leading to NFTs [317]. Synaptic vesicles and exocyst cycling, and receptor targeting may be impacted during Tau pathology. Postsynaptic roles in Tau pathophysiology have been related to AMPAR or NMDAR localisation, trafficking or functioning [92, 190, 200, 313, 434, 465]. Hoover et al. [190] showed, using rTgP301L mice, that Tau mutation or hyperphosphorylation impaired trafficking or anchoring of AMPARs and NMDARs. Multiple indirect mechanisms of NMDAR or AMPAR-dependent impairment have been shown through changes to import Fyn kinase, PSD95, and KIBRA proteins into post-synaptic compartments [200, 447, 465].

Based on recent evidence from the literature, we hypothesise that Tau serves as a scaffold to bind the cytoskeleton and to regulate its interactions with key synaptic targets, particularly in coordinating CME at both the pre- and post- synaptic compartments. A similar role for Aβ in CME and clathrin-dependent membrane and protein trafficking pathways, which is known to affect synaptic vesicle endocytosis and exocytosis, has already been posited in AD [223, 346, 485], in schizophrenia and bipolar disorder [396]. Aβ42 oligomers are known to directly interact with Syntaxin 1a [485] Synaptophysin [387], or indirectly interfere with dynamin through NMDAR activation [222, 224], and Synapsin1 [274, 292, 342]. Although we have listed many possible pathways by which Tau may mediate its role at the synapse based on its binding ability, not all of these may have functional relevance or be directly related to AD pathology. These pathways may however highlight the intricacies of the dysfunction that may occur, or at least show the complexity of the etiology and progression of AD.

## Outstanding questions

Does Tau have physiological roles in the pre- and/or post-synaptic compartments for pathways related to vesicle cycling and protein trafficking for plasticity, or is its localisation in synaptic compartments purely pathological?

What is the phenomenon that causes the conversion of monomeric to multimeric Tau species? Does Tau aggregation impair any physiological roles of synaptic Tau and if so how and at what point(s) during the aggregation pathway? Is pathology to pathways involving physiological Tau directly responsible for memory impairment seen in AD?

At what stage does pathological phosphorylation of Tau occur and how does this deter from physiological phosphorylation pathways and normal function?

Is the presence of Tau at different locations in cells due to internal Tau translocations or due to Tau being released into the extracellular space and its re-uptake by neighbouring neurons? And therefore, how does the extracellular environment and Tau uptake into the endo/lysosomal pathway affect Tau location and pathology?

Of the proteins discussed in this review as being capable of binding Tau, which of these interactions have functional roles inside of neurons? Are these interactions affected by multimeric Tau and could they be therapeutically targeted?

## Supplementary Information

**Additional file 1**. Supplementary Table 1. The proteins discussed in this article that are capable of binding to Tau and have involvement in synaptic pathways.

## Abbreviations

Aβ: Amyloid-beta
AD: Alzheimer’s disease
AMPAR: α-Amino-3-hydroxy-5-methyl-4-isoxazolepropionic acid receptor
CME: Clathrin mediated endocytosis
LOAD: Late onset Alzheimer’s disease
LTD: Long-term depression
LTP: Long-term potentiation
MAPT: Microtubule-associated protein Tau
MTB: Microtubule binding repeat region
NFT: Neurofibrillary tangle
NMDAR: N-methyl-D-aspartate receptor
UPS: Ubiquitin proteasome system
