# Supplementary material for: Synaptic tau: A pathological or physiological phenomenon?
Source: Acta Neuropathol Commun. 2021 Sep 9;9:149. doi: 10.1186/s40478-021-01246-y (PMC8428049; doi:10.1186/s40478-021-01246-y)
Supplement: Supplementary file 1 — Additional file 1. Supplementary Table 1. The proteins discussed in this article that are capable of binding to Tau and have involvement in synaptic pathways. [file 40478_2021_1246_MOESM1_ESM.docx]

**Supplementary**

Table 1. The proteins discussed in this article that are capable of binding to Tau and have involvement in synaptic pathways.

| **Protein** | **Function(s)** | **Publication** | **Preferential binding Isoform** (Liu *et al.*, 2016) |
| --- | --- | --- | --- |
| 14-3-3ζ | Regulatory protein | (Hernández, Cuadros and Avila, 2004; Liu *et al.*, 2016) | 2N |
| Actin | Cytoskeleton | (Fulga *et al.*, 2006; He *et al.*, 2009; Hsueh, 2012) | 1N |
| Adducin | Membrane scaffold | (Liu *et al.*, 2016) | 0N |
| Alpha-synuclein | Presumed endo/exocytosis | (Liu *et al.*, 2016) | 0N |
| Amyloid precursor protein | Membrane protein | (Islam and Levy, 1997) |  |
| AP2 | Endocytosis | (Liu *et al.*, 2016) | 1N |
| Apolipoprotein E | Membrane protein | (Liu *et al.*, 2016) | 2N |
| Bin-1 | Endo/exocytosis | (Sottejeau *et al.*, 2015; Lasorsa *et al.*, 2018) |  |
| Calcineurin | Signalling | (Liu *et al.*, 2016) |  |
| Calmodulin | Signaling | (Sobue *et al.*, 1981; Liu *et al.*, 2016) | 1N |
| CaMKII | Signalling | (Sironi *et al.*, 1998) |  |
| CaMKv | Signalling | (Liu *et al.*, 2016) | 0N |
| Clathrin | Endocytosis | (Liu *et al.*, 2016) | All equal |
| Cofilin | Cytoskeleton | (Liu *et al.*, 2016) | All equal |
| Dynamin-1 | Endocytosis | (Liu *et al.*, 2016) | All equal |
| Fyn kinase | Signalling | (Lee *et al.*, 2004) |  |
| GAP-43/neuromodulin | Signalling | (Liu *et al.*, 2016) | 1N |
| Lrp1 | Apolipoprotein E receptor | (Cooper *et al.*, 2020) |  |
| Myelin Binding Protein | Myelin structuring | (Liu *et al.*, 2016) | 1N |
| Neurochondrin | Signalling | (Liu *et al.*, 2016) | 2N |
| Neurogranin | Signalling | (Liu *et al.*, 2016) | 0N |
| NSF | Endocytosis | (Liu *et al.*, 2016) | 1N |
| Pyk2 | Signalling | (Li and Götz, 2018) |  |
| Septin-7 | Cytoskeletal | (Liu *et al.*, 2016) | 2N |
| Sorl1 | Apolipoprotein E receptor | (Cooper *et al.*, 2020) |  |
| SV2A | Synaptic vesicle protein | (Liu *et al.*, 2016) | 0N |
| Synapsin I | Synaptic vesicle protein | (Liu *et al.*, 2016) | 0N |
| Synapsin II | Synaptic vesicle protein | (Liu *et al.*, 2016) | All equal |
| Synaptobrevin/  VAMP | Synaptic vesicle protein | (Liu *et al.*, 2016) | 2N |
| Synaptogyrin-3 | Synaptic vesicle protein | (Liu *et al.*, 2016; McInnes *et al.*, 2018) | 0N |
| Synaptophysin | Synaptic vesicle protein | (Liu *et al.*, 2016) | All equal |
| Synaptotagmin-1 | Synaptic vesicle protein | (Liu *et al.*, 2016) | 2N |
| Syntaxin-1B | Exocytosis | (Liu *et al.*, 2016) | 2N |
| Transferrin | Fe^3+^-binding/endocytosis | (Liu *et al.*, 2016) | 2N |
| Tropomyosin-1 | cytoskeletal | (Liu *et al.*, 2016) | 1N |
| v-ATPase | Vesicle protein | (Liu *et al.*, 2016) |  |
